# Peritoneal NK cells are responsive to IL-15 and percentages are correlated with outcome in advanced ovarian cancer patients

**DOI:** 10.18632/oncotarget.26199

**Published:** 2018-10-05

**Authors:** Janneke S. Hoogstad-van Evert, Ralph J. Maas, Jolien van der Meer, Jeannette Cany, Sophieke van der Steen, Joop H. Jansen, Jeffrey S. Miller, Ruud Bekkers, Willemijn Hobo, Leon Massuger, Harry Dolstra

**Affiliations:** ^1^ Department of Obstetrics and Gynecology, Radboud University Medical Center, Nijmegen, The Netherlands; ^2^ Department of Laboratory Medicine, Laboratory of Hematology, Radboud University Medical Center, Nijmegen, The Netherlands; ^3^ Department of Medicine, Division of Hematology, Oncology, and Transplantation, University of Minnesota, Minneapolis, Minnesota, USA

**Keywords:** natural killer cells, ovarian cancer, IL15, ascites, survival

## Abstract

The demonstration that ovarian carcinoma (OC) is an immunogenic disease, opens opportunities to explore immunotherapeutic interventions to improve clinical outcome. In this regard, NK cell based immunotherapy could be promising as it has been demonstrated that OC cells are susceptible to killing by cytokine-stimulated NK cells. Here, we evaluated whether percentage, phenotype, function and IL-15 responsiveness of ascites-derived natural killer (NK) cells is related to progression-free survival (PFS) and overall survival (OS) of advanced stage OC patients. Generally, a lower percentage of NK cells within the lymphocyte fraction was seen in OC ascites (mean 17.4 ± 2.7%) versus benign peritoneal fluids (48.1 ± 6.8%; *p* < 0.0001). Importantly, a higher CD56+ NK cell percentage in ascites was associated with a better PFS (*p* = 0.01) and OS (*p* = 0.002) in OC patients. Furthermore, the functionality of ascites-derived NK cells in terms of CD107a/IFN-γ activity was comparable to that of healthy donor peripheral blood NK cells, and stimulation with monomeric IL-15 or IL-15 superagonist ALT-803 potently improved their reactivity towards tumor cells. By showing that a higher NK cell percentage is related to better outcome in OC patients and NK cell functionality can be boosted by IL-15 receptor stimulation, a part of NK cell immunity in OC is further deciphered to exploit NK cell based immunotherapy.

## INTRODUCTION

Because ovarian carcinoma (OC) is generally asymptomatic until ascites or metastases beyond the ovaries have developed, patients are often diagnosed in advanced stage. Moreover, the presence and progression of ascites is associated with dismal prognosis and poor quality of life [1]. Current therapy consists of debulking surgery combined with platinum/taxane chemotherapy, but the majority of patients develop a recurrence within 3 years. Especially, for women with advanced stage disease the prognosis is dismal, and despite therapeutic advances the 5-year survival is only 28% [2].

Since many studies demonstrated that OC is an immunogenic disease, further research is needed to explore the opportunities of immunotherapeutic interventions to improve clinical outcome. In this regard, OC ascites is an attractive source to study immune cell function in patients because tumor cells and immune cells are both present. Furthermore, ascites contains a variety of immunosuppressive cellular and soluble components that influence the function of tumor-targeting lymphocytes [3–8]. Several studies showed that presence of tumor-infiltrating lymphocytes positively correlated with survival in cancer patients [9–16]. While the importance of CD8+ T cell infiltration has been clearly demonstrated, the role of infiltrating innate natural killer (NK) cells remains unclear. Interestingly, it was reported that CD103+ tumor-infiltrating NK cells often co-infiltrate with CD8+CD103+ T cells, yet the contribution of NK cells to improving outcome is difficult to assess [11]. Therefore, more research is required to decipher the role of NK cell immunity in OC patients.

NK cells are activated against neoplastic cells through a balance of activating and inhibitory receptors [17, 18]. Epidemiological research has shown that low NK cell activity is associated with increased cancer risk in humans [19]. For OC patients, decreased functionality of ascites-derived NK cells has been observed [20], which could be partially attributed to the low expression of various activating NK cell receptors including CD16, DNAM-1 and NKp30 [8, 21, 22]. Similarly, ascites-derived T cells are rather inactive, though proliferation and functionality can be partially restored by cytokine stimulation [23]. NK cells from ascites also exhibit low cytotoxic efficacy, which could be reinvigorated by IL-2 or IL-15 [24]. In this regard, Felices *et al.* recently reported that the, ALT-803, a fusion protein complex of IL-15 variant (N72D) bound to sushi domain of IL-15Rα fused to IgG1 Fc, potently enhanced the function of ascites-derived NK cells and healthy donor peripheral blood NK cells exposed to ascites fluid [25]. Most importantly, many studies demonstrated that OC cells are susceptible to killing by cytokine-stimulated NK cells [26–41].

In this study, we characterized NK cell percentage, phenotype and functionality in ascites of advanced OC patients in relation to clinical outcome, and investigated their responsiveness to IL-15 receptor mediated stimulation. We observed that a higher CD56+ NK cell proportion within the ascites lymphocyte fraction was associated with better progression free survival (PFS; *p* = 0.01) and overall survival (OS; *p* = 0.002) in OC patients. Furthermore, we demonstrated that the cytolytic function of ascites-derived NK cells can be effectively reinvigorated with either monomeric IL-15 or the IL-15 superagonist fusion complex, ALT-803. These findings indicate that boosting NK cell expansion and functionality by immunotherapeutic strategies could improve survival in OC patients.

## RESULTS

### Patient cohort characteristics

For this study, we selected ascites fluid samples collected at diagnosis or first surgery of patients with stage IIIc or IV high-grade serous papillary OC. The mean age of the selected OC patient cohort (*n* = 20) was 64 ± 8.8 years and 48 ± 8.1 years for the benign gynecological disorder control group (*n* = 10). The median OS and PFS of the OC patient cohort at time of analysis was 19 months and 6 months, respectively. Based on the median OS, the patient cohort was divided in two groups: i.e. poor survival group (*n* = 10) with an OS of less than 19 months and good survival group (*n* = 10) with an OS of more than 19 months (Table 1). The OS and PFS in the good survival group were 32.9 ± 11.2 and 19.7 ± 16.4 months, respectively. Whereas the OS and PFS in the poor survival group was only 10.3 ± 4.4 and 3.2 ± 2.3 months, respectively. Further characteristics of the two OC patient groups are shown in Table 1. Patients in the good survival group were younger and were less often postmenopausal. In both groups, half of the OC patients were treated with primary surgery, and half with neo-adjuvant chemotherapy. CA-125 levels were higher in the good survival group.

**Table 1:** Patient characteristics

	Characteristics	Good survival (*n* = 10)	Poor survival (*n* = 10)
**Baseline**	Age mean (SD)	57.2 (8.4)	69.0 (11.4)
	CA-125 mean (95% CI)	1532 (641–2423)	1268 (199–2337)
	Postmenopausal	70%	90%
**Treatment**	Neoadjuvant Chemotherapy	50%	60%
	Primary surgery	50%	40%
**Debulking**	Complete Debulking	20%	20%
	(Sub-) Optimal Debulking	80%	70%
	No Debulking	0%	10%
**Survival**	PFS mean (SD)	19.7 (16.4)	3.2 (2.3)
	OS mean (SD)	32.9 (11.2)	10.3 (4.4)

### High peritoneal NK cell proportion within the lymphocyte fraction is associated with better survival of OC patients

**Figure 1:**
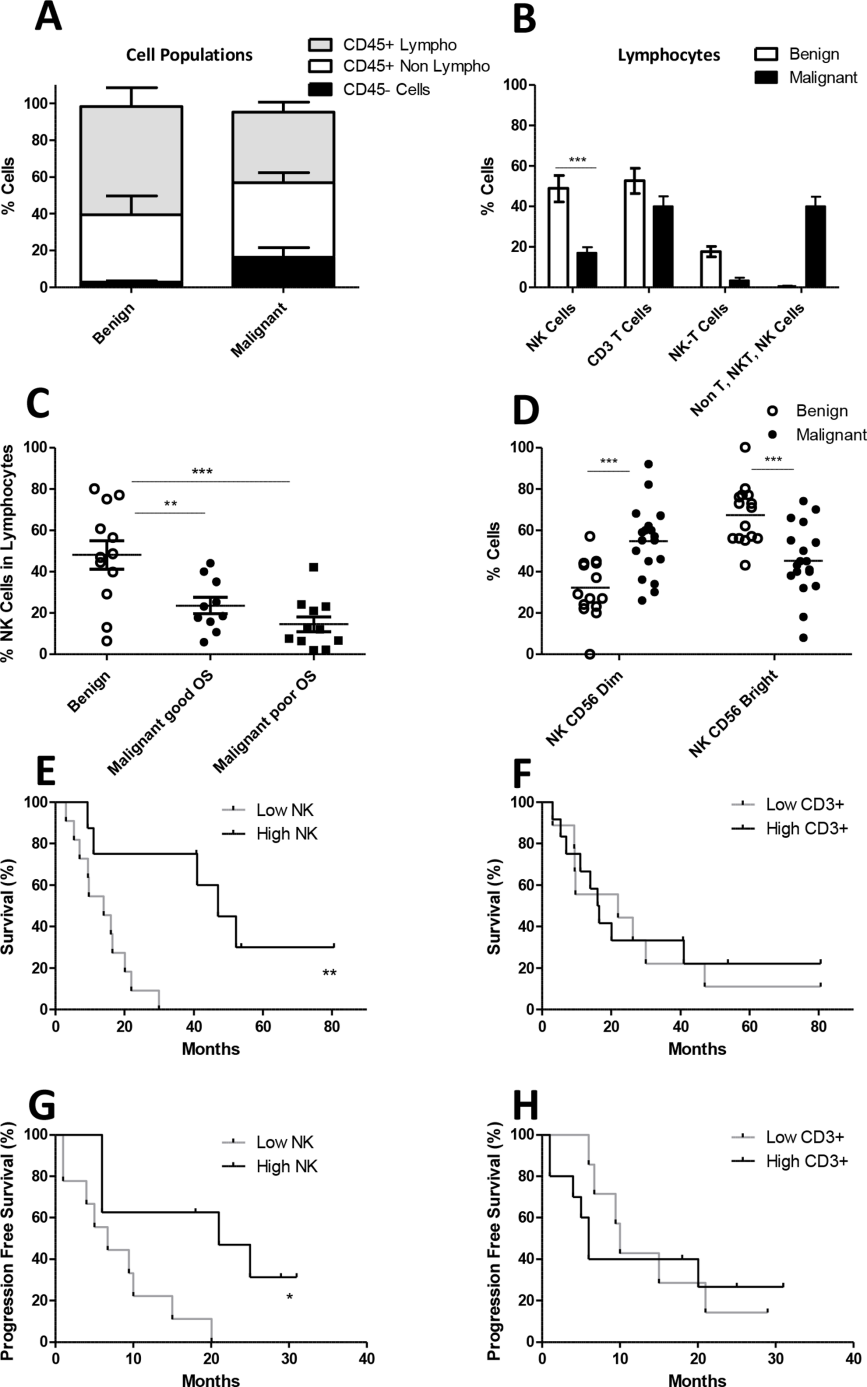
NK, NKT and T cell percentage in benign ascites and ascites from ovarian cancer patients (**A**) Fraction of CD45+ lymphocytes (white), CD45+ non-lymphocytes (grey) and CD45- cells (black) cell populations within peritoneal fluid of benign compared to malignant ovarian cancer patients, based on flow cytometric analysis of CD45 expression and forward/side scatter. (**B**) Percentage of NK cells, T cells, NKT cells and other lymphocytes within benign and malignant ascites. The percentage NK cells within the lymphocyte population is significantly different, two tailed *T*-test *p* < 0.0001. (**C**) Within the lymphocyte population the percentage of NK cells is depicted. The group of malignant ovarian carcinoma ascites patients is divided into good and poor survival based on the median survival of the analyzed patient cohort (*n* = 20). (**D**) The NK cell population is subdivided based on CD56 bright and CD56 dim cells. (**E**) Overall survival curve of OC patients groups with low and high CD56+ NK cell percentages in ascites. (**F**) Overall survival curve of OC patients groups with low and high CD3+ T cell percentages in ascites. (**G**–**H**) Progression free survival curves for low and high CD56+ NK cell and CD3+ T cell percentages in ascites. Error bars represent mean + SEM. When 2 groups were compared the Student *T*-test was used whereas a one-way ANOVA with Bonferroni correction was performed when comparing 3 groups. ^***^p = 0.001.

First, we assessed the percentages of NK, NKT and T cells within the lymphocyte fraction in cryopreserved ascites samples of the selected OC patients by flow cytometry and compared those with peritoneal fluid of 10 patients with a benign gynecological disorder. Within the total cell fraction OC ascites contained 38.8 ± 24.8% lymphocytes, 40.5 ± 24.7% CD45+ non-lymphocytes and 16.4 ± 23.5% CD45- non-hematopoietic tumor cells, and the benign samples contained 58.7 ± 40.4% lymphocytes and 36.5 ± 34.1% non-lymphocytes within CD45+ leucocytes (Figure 1A). Within the lymphocytes a significantly lower CD3-CD56+ NK cell percentage was seen in OC patient ascites (mean 17.1 ± 2.7%) compared to benign peritoneal fluid (48.1 ± 6.8%, *p* < 0.0001; Figure 1B). Furthermore, lower CD3+ T cell and CD3+CD56+ NKT cell percentages were observed within the lymphocyte population in OC patient ascites. The population of non T-, non-NKT, non-NK cells in the lymphocyte gate, presumably B cells, was more prominent in the malignant samples (Figure 1B). Notably, the group of OC patients with poor survival had 14.5 ± 3.6% NK cells versus 23.6 ± 4.0% in the patients with good survival (Figure 1C). In addition, we observed a significant shift in the CD56^dim/bright^ ratio in OC patients in comparison to peritoneal fluid of patients with a benign gynecological disorder (Figure 1D). Generally, in healthy donor blood around 90% cytotoxic CD56^dim^ and 10% regulatory CD56^bright^ cells are present [42]. In contrast, in the benign ascites samples we found 32.4 ± 3.7% NK^dim^ cells and 67.5 ± 3.7% CD56^bright^ cells, respectively. In OC patient ascites, however, the ratio was more in favor of the cytotoxic CD56^dim^ population with 54.7 ± 4.0% CD56^dim^ and 45.4 ± 4.0% CD56^bright^ cells, compared to the benign peritoneal fluids (Figure 1D).

Next, we divided the OC patients in two groups based on the median ascites NK cell percentage within the lymphocyte fraction into high (mean 31.4 ± 9.4%) and low (mean 8.9 ± 4.6%) percentage groups. We observed that both OS (*p* = 0.002, hazard ratio = 5.7) and PFS (*p* = 0.01, hazard ratio = 4.7) were significantly better in OC patients with a high peritoneal NK cell percentage versus patients with a low NK cell percentage in the lymphocyte fraction (Figure 1E and 1G). Notably, this relationship was not observed for CD3+ T cell percentages in ascites (Figure 1F and 1H). Interestingly, in the high NK group three patients are still alive after a follow-up of ≥50 months. Altogether, these data indicate that the CD56+ NK cell percentage within the lymphocyte fraction in ascites fluid of OC patients is positively correlated with clinical outcome.

### Ascites-derived NK cells of poor survival ovarian carcinoma patients exhibit low expression levels of activating receptors

**Figure 2:**
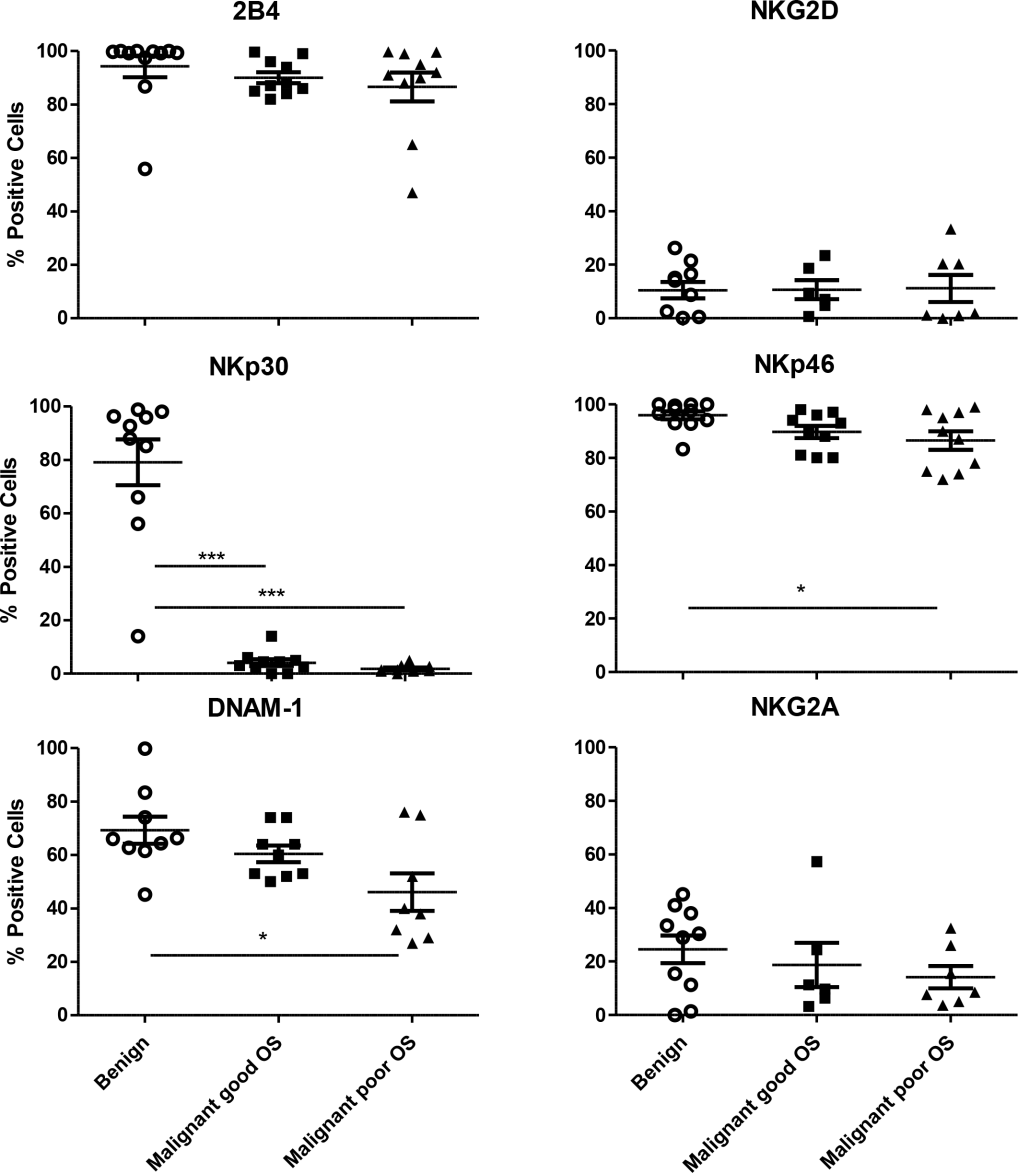
Expression of activating receptors on CD45+CD3-CD56+ NK cells Percentage positive 2B4, NKG2D, NKp46, NKp30, DNAM-1 and NKG2A NK cells of CD56+ NK cells in benign and malignant peritoneal fluid. The group of malignant ovarian carcinoma ascites patients is divided into lower than median overall survival and higher than median overall survival. Error bars represent mean + SEM. One way ANOVA with Bonferroni correction was performed when comparing the groups. ^*^= *p* < 0.05, ^***^= *p* < 0.001.

Next to the percentage of NK cells in ascites, we studied whether survival of OC patients was associated with differential expression of NK cell activating receptors. Hereto, we performed flow cytometry analysis on the ascites-derived NK cells of the selected patient cohort and benign ascites controls (Figure 2). While 2B4 had equally high positivity on both benign and malignant peritoneal fluid NK cells, NKG2D was low to undetectable on these NK cells. Remarkably, NKp30 was almost absent on NK cells in malignant samples (mean 3%), whereas it was significantly higher on NK cells from benign samples (mean 79%). NKG2A can suppress activation, and shows no significant differences between benign and malignant samples. Moreover, NKp46 and DNAM-1 were significantly lower expressed on NK cells in malignant samples, especially in patients with a poor OS. Together, these data indicate that ascites NK cells of OC patients with poor survival have significantly lower expression of activation receptors on their surface.

### Ascites-derived NK cells possess equal cytotoxic function as peripheral blood NK cells from healthy donors

**Figure 3:**
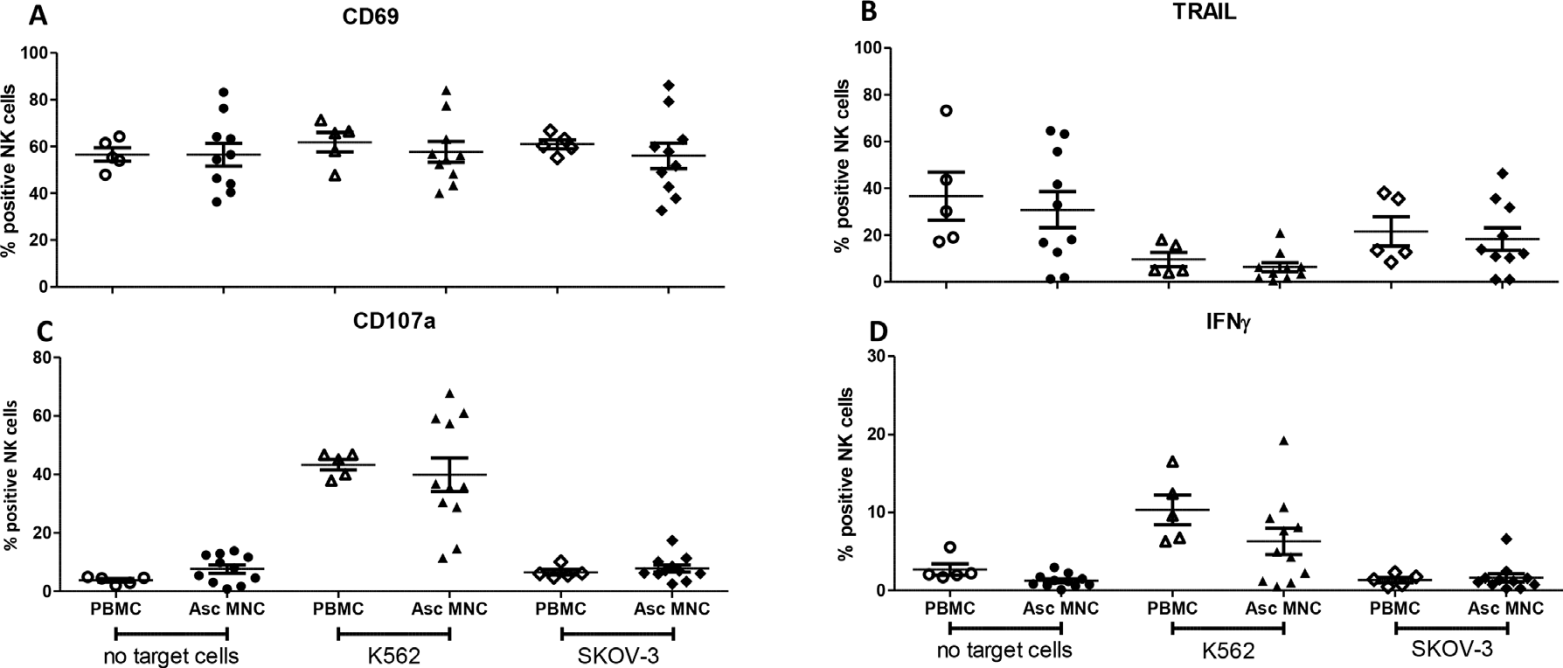
Degranulation assay comparing NK cells in healthy donor (HD) peripheral blood mononuclear cells (PBMCs) with ascites mononuclear cells (MNCs) Percentage CD56+ NK cells positive for (**A**) CD69, (**B**) TRAIL, (**C**) CD107a, (**D**) IFN-γ, after 4 h stimulation with no target cells, K562 or SKOV-3 tumor cells. Error bars represent mean + SEM. Open symbols depict HD PBMCs, closed symbols depict ascites MNCs. One way ANOVA with Bonferroni correction was performed when comparing the groups.

Next, we addressed the functional activity of peritoneal fluid NK cells of OC patients in comparison to peripheral blood (PB)-NK cells from healthy donors. Here, we cultured MNCs from ascites of OC patients or PB of healthy controls overnight in the presence of IL-15, whereupon total cells were challenged for 4 hours with either K562 (control), SKOV-3 OC or without tumor cells and subsequently analyzed by flow cytometry. We examined expression of the activation markers CD69 and TRAIL, the degranulation of NK cells using CD107a, and intracellular IFN-γ positivity. Both ascites and control NK cells showed high CD69 expression, while TRAIL levels decreased upon tumor challenge (Figure 3A and 3B). Furthermore, ascites-derived NK cells were capable of exerting a CD107a degranulation and IFN-γ response against K562 cells (Figure 3C and 3D). However, reactivity of ascites-derived NK cells greatly varied between different OC patients, therefore no significant differences were observed as compared to healthy donor NK cells. Notably, for both OC ascites and healthy donor NK cells the response against SKOV-3 OC cells was poor. Together, these data demonstrate that OC ascites-derived NK cells have equivalent degranulation and IFN-γ secretion capacity as healthy donor PB-NK cells, yet responsiveness against SKOV-3 OC cells is limited.

### Functionality of ascites NK cells can be effectively boosted by IL-15 or ALT-803 stimulation

**Figure 4:**
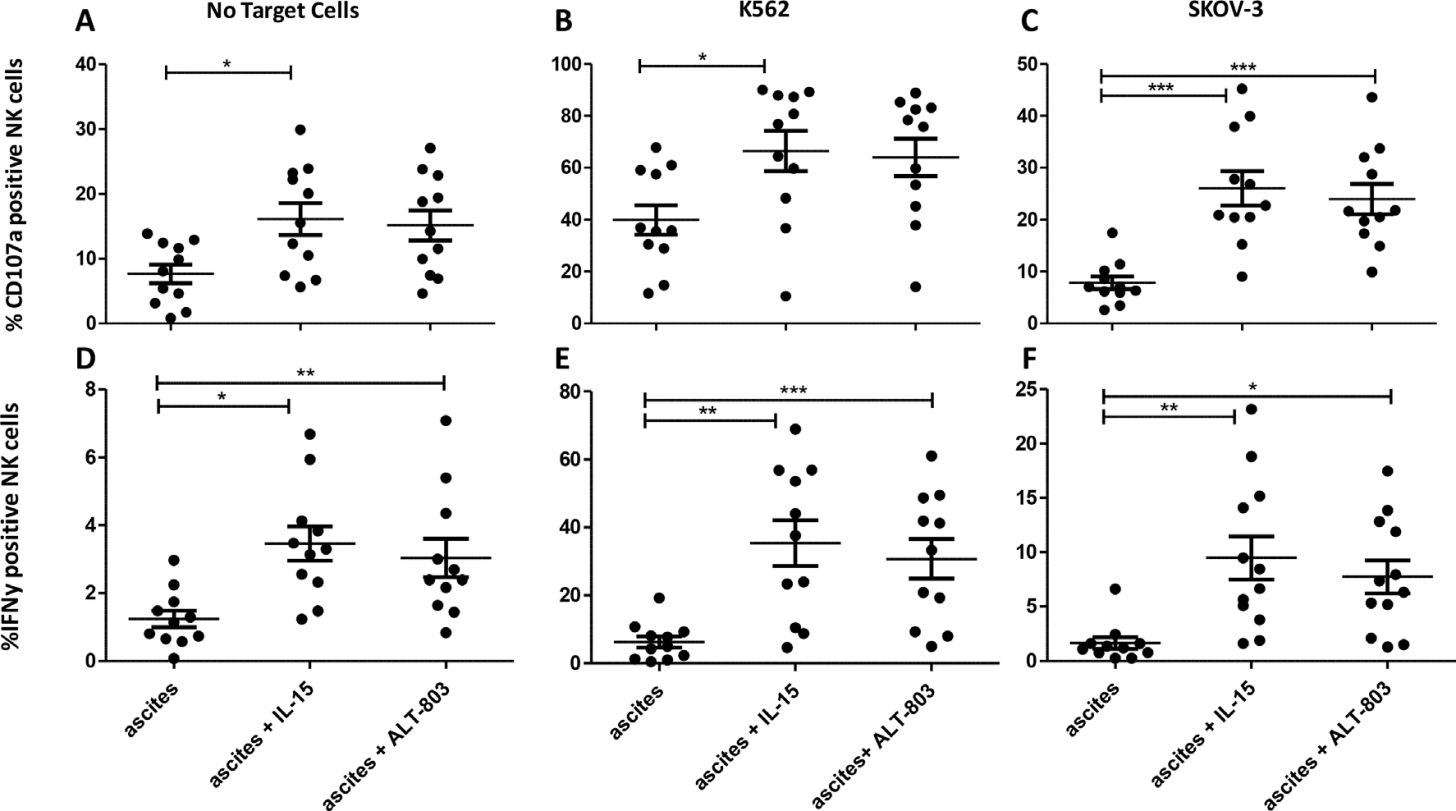
Degranulation assay comparing ascites CD56+ NK cells with and without monomeric IL-15 or ALT-803 stimulation (**A**–**C**) Percentage CD56+ NK cells positive for CD107a after 4 h co-culture with no stimulation (A), K562 cells (B) or SKOV-3 cells (C). (**D**–**F**) Percentage CD56+ NK cells positive for IFN-γ after 4 h co-culture with no stimulation (D), K562 cells (E) or SKOV-3 cells (F). Error bars represent mean + SEM. One way ANOVA with Bonferroni correction was performed when comparing the groups. ^*^ = *p* < 0.05, ^**^ = *p* < 0.01, ^***^ = *p* < 0.001.

To improve peritoneal NK cell reactivity against OC, we investigated whether boosting with monomeric recombinant human IL-15 or the human IL-15 superagonist fusion complex, ALT-803. Interestingly, IL-15 receptor-mediated stimulation already enhanced CD107a expression and IFN-γ secretion capacity of the NK cells in the absence of tumor challenge (Figure 4A and 4D). Most importantly, ascites-derived NK responsiveness against K562 and especially SKOV-3 OC cells could be potently augmented by IL-15 or ALT-803 stimulation (*p* < 0.001 for CD107a and *p* < 0.01 for IFN-γ; Figure 4B, 4C, 4E and 4F). These data demonstrate that the function activation of NK cells in ascites can be efficiently rescued with IL-15 or ALT-803.

## DISCUSSION

NK cells are activated against cancer cells via activating and inhibitory receptors, and the balance in these signals determines the magnitude of their activity. [17, 18] Epidemiological research has shown that low NK cell activity is associated with increased cancer risk in humans [19]. Moreover, NK cells have been identified to play a role in tumor surveillance due to enhanced surface expression of ligands for activating receptors on tumor cells by the DNA damage response [43]. Together these studies underscore the contribution of NK cell function in anti-cancer immunity. However, there is limited data on the contribution of NK cell immunity on the clinical outcome of women with ovarian carcinoma. In the present study, we found that the percentage of CD56+ NK cells within the lymphocyte fraction in ascites fluid is related to OS and PFS, and that ascites-derived NK cells have lower expression of activation markers than benign peritoneal fluid NK cells. Although, ascites-resident NK cells have poor reactivity against SKOV-3 OC cells (similarly as PB-NK cells) they can be effectively boosted by IL-15 receptor mediated stimulation.

In our current study, OC patients were randomly selected from our ascites biobank. Nevertheless, as large amounts of ascites MNCs was required for these studies, we thereby selected automatically a group of patients with a relatively poor prognosis. A remarkably high percentage of these patients did not undergo complete debulking surgery, however since rates of complete debulking were comparable in both the poor and good OS groups, we believe that the impact of incomplete debulking on our NK cell correlative results is limited. Notably, the OS of our patient cohort is comparable to a large national cohort [44]. Although there were age differences between the benign control and the OC groups we do not expect impact on our findings, as it has been reported for PB that NK cell percentages are very stable and do not change with age [45].

Notably, a higher NK cell percentage within the lymphocyte fraction was found in peritoneal fluid of benign control subjects than in ascites of OC patients. Often peritoneal fluid of healthy individuals is used in endometriosis research, and also in this disease a significantly lower percentage of NK cells in peritoneal fluid has been reported [46]. In OC the only relation between clinical outcome and NK cells in ascites was described by Dong *et al.*, and they reported CD16 positive cells to be associated with poorer outcome of OC patients. Unfortunately, CD56 expression was not evaluated in their report [12]. Our paper is the first report to show a relationship between the CD56+ NK cell proportion within the ascites lymphocyte fraction and clinical outcome parameters. While the importance of CD8+ T cell infiltration within OC tumors has been clearly demonstrated, and also CD3+ T cells in ascites have been found to correlate with better outcome [47, 48], a relation between CD3+ T cell percentage and survival was not observed in our cohort. Besides percentages, absolute numbers of infiltrating lymphocytes is an important parameter for correlation with clinical outcome. However, as volume and cellular density in ascites differed greatly between OC patients, we believe that the percentage within the lymphocyte population was the most objective way to compare cell populations. For future research, it would be interesting to investigate how ascites NK cells differ from blood NK cell numbers, phenotype and function in the same OC patient.

It has been previously reported that OC ascites NK cells have been described to exhibit lower expression of the activating markers NKp30, NKp46, NKG2D and DNAM-1 compared to healthy donor PB-NK cells [8, 49]. In our study, we observed the same significantly lower expression of NKp30, NKp46 and DNAM-1 on OC ascites-derived NK cells compared to NK cells from benign peritoneal fluid. However, the relationship between NKG2D and prognosis was not found in our dataset. Interestingly, we observed a significant lower NK cell expression of NKp30 in poor prognosis versus good prognosis ascites samples, suggesting that NKp30 expression changes in the poor prognosis OC environment. The same has been reported in acute myeloid leukemia, where NKp30 is proposed as a prognostic biomarker based on its low expression on NK cells in poor prognosis patients [50]. Further validation in a larger patient cohort is required to establish prognostic biomarkers in OC patients.

In our functional studies, ascites-derived NK cells demonstrated equal degranulation and cytokine secretion potential as healthy donor PB-NK cells. This corresponds with findings by Felices *et al.* who showed comparable levels of CD107a after stimulation in the presence or absence of tumor cells [25]. In contrast, other reports demonstrated that ascites-derived NK cells were dysfunctional, with decreased CD16 expression and low cytotoxic capacity [6, 51, 52]. Here, we showed that ascites-derived NK cells were highly capable of recognizing K562 tumor cells, but exhibited poor SKOV-3 OC cell reactivity. Most importantly, we demonstrated that stimulation with IL-15 or ALT-803 could reinvigorate NK cell degranulation and IFN-γ production, especially against SKOV-3 OC cells. Although our *ex vivo* studies did not show any difference between IL-15 and ALT-803, ALT-803 is likely more potent in long-term assays and *in vivo* because of its longer half-life [53, 54].

Concluding, this report shows a significant association between the percentage and phenotype of NK cells within the lymphocyte fraction in peritoneal fluid and survival of OC patients. Moreover, we demonstrated that peritoneal NK cell reactivity against OC tumor cells can be efficiently boosted by IL-15 receptor-mediated stimulation. The relationship between availability of NK cells in the abdominal cavity and the potentiating effect of IL-15 indicates that intraperitoneal NK cell adoptive transfer combined with IL-15 administration could be an interesting new therapy for OC patients to improve outcome. Currently, a phase 1 clinical trial testing intraperitoneal ALT-803 therapy in OC patients is enrolling patients in the US (NCT0354909) and a phase 1 clinical trial on intraperitoneal NK cell therapy exploiting CD34+ progenitor-derived NK cells is open in the Netherlands (NCT03539406). By demonstrating that high NK cell percentages are associated with better outcome in OC patients and peritoneal NK cell functionality can be boosted by IL-15 receptor stimulation, a part of NK cell immunity in OC is further deciphered to exploit NK cell based immunotherapy in these poor prognosis patients.

## MATERIALS AND METHODS

Ascites fluid samples were prospectively collected at diagnosis or first surgery of patients with stage IIIc or IV high-grade serous papillary OC between January 2009 and January 2013 at the Radboud University Medical Center (RUMC). Study approval was given by the Regional Committee for Medical Research Ethics (CMO 2013-516) and performed according to the Code for Proper Secondary Use of Human Tissue (Dutch Federation of Biomedical Scientific Societies, www.federa.org). Ascites was filtered using a 100 μm filter, washed and MNCs were isolated by Ficoll-Hypaque density gradient centrifugation. Subsequently, obtained cells were cryopreserved and stored in liquid nitrogen until use. From this biobank we randomly selected ascitic cell samples from 20°C patients. For the benign control group, samples were collected at benign gynecological surgeries. Main indication for diagnostic laparoscopy was abdominal pain, samples were included only if pathological findings were absent. Detection of cysts, endometriosis and adhesions at laparoscopy were exclusion criteria. These benign samples were processed and analyzed on the day of surgery. Medical records were retrospectively reviewed and relevant clinical and pathology data were extracted. Time of diagnosis was considered to be the date of the primary surgical procedure. Time from diagnosis to death was calculated for OS. PFS was calculated as time of last chemotherapy till diagnosis of biochemical or radiologic recurrence. Median survival was expressed in months.

Patients were divided in groups for Figure 1C and Figure 2 based on median overall survival, poor survival are the patients with an overall survival of less than the median (19 months), good survival are the patients with an overall survival of more than 19 months.

For Figure 1E and 1G patients were divided based on median NK cell frequency, for Figure 1F and 1H groups are divided by median CD3 cell frequency.

### Flow cytometry

MNCs were stained with labeled antibodies, CD3 ECD (Biolegend), CD45 Krome Orange (R&D systems), CD56 PE-Cy5 (Biolegend), CD16 APC-Cy7 (Biologend), CD326 PerCPCy5.5 (Biolegend). Phenotypic analysis was performed using DNAM-1 FITC (Becton Dickinson), 2B4 FITC (Biolegend), NKG2A APC (Beckman Coulter), NKG2D APC (Biolegend), NKp30 PE (Biolegend) and NKp46 PE(Biolegend), isotype controls for IgG1 and IgG2a, (all Biolegend). Dead cells were stained with 1:1000 diluted sytox blue (Life Technologies; Invitrogen). Flow cytometry analysis was performed on a Gallios flow cytometer from Beckman Coulter. Analysis was done in Kaluza 1.5 (Beckman Coulter). Gating strategy is shown in Supplementary Figure 1.

### K562 and SKOV-3 cell lines

OC cell line SKOV-3 was cultured in Roswell Park Memorial Institute medium (RPMI 1640; Gibco) medium supplemented with 10% Fetal Calf Serum (FCS; Integro). The chronic myeloid leukemia cell line K562 was cultured in Iscove's Modified Dulbecco's Medium (IMDM) with 10% FCS.

### Functional assay (CD107a and IFN-γ)

After thawing ascites MNCs or PBMCs were cultured overnight with 1 nM IL-15 (Immunotools), 1 nM ALT-803 (Altor Bioscience) or without cytokine support in IMDM with 10% FCS and 1% penicillin/streptomycin (p/s). Subsequently, 1 × 10^6^ cells were co-cultured with 0.5 × 10^6^ K562 cells, 0.5 × 10^6^ SKOV-3 cells or without target cells for 4 hours in IMDM with 10% FCS and 1% p/s and anti-CD107a PE-Cy7 (Biolegend) in a 24-well plate. After 1 h of co-culture, brefeldin A (BD) was added. Finally, cells in each well were gently resuspended and stained with labeled antibodies, CD56 BV510 (Biolegend), CD45 AF700 (Invitrogen), CD3 ECD (Beckman Coulter), CD69 BV421 (Biolegend) or isotype IgG1BV421 (BD biosciences), and TRAIL APC or isotype IgG1 APC (both Biolegend). Dead cells were stained with 1:1000 in PBS diluted eFluor780. Next, cells were fixed, permeabilized and stained with anti-IFN-γ or isotype IgG1 FITC (BD biosciences) and anti-perforin or isotype IgG2b PE (Biolegend). Flow cytometry acquisition was performed on a Gallios flow cytometer from Beckman Coulter. Analysis was done in Kaluza 1.5 (Beckman Coulter).

### Statistical analysis

Statistical analysis was performed in Graphpad Prism software package version 5.03. Flow cytometry data was expressed as percentage positive cells. Data were analyzed using two-way ANOVA for group comparison or one-way ANOVA with Bonferroni post-hoc correction if more than two groups were compared. Unpaired *T*-tests were performed for comparison of two single groups, as indicated. Differences were considered significant when the *p* value was < 0.05. Survival curves were analyzed by Log rank (Mantel-Cox) test.

## SUPPLEMENTARY MATERIALS FIGURE


