# Supplementary material for: Peritoneal NK cells are responsive to IL-15 and percentages are correlated with outcome in advanced ovarian cancer patients
Source: Oncotarget. 2018 Oct 5;9(78):34810–20. doi: 10.18632/oncotarget.26199 (PMC6205176; doi:10.18632/oncotarget.26199)
Supplement: Supplementary file 1 [file oncotarget-09-34810-s001.pdf]

Peritoneal NK cells are responsive to IL-15 and percentages are correlated with outcome in advanced ovarian cancer patients

SUPPLEMENTARY MATERIALS

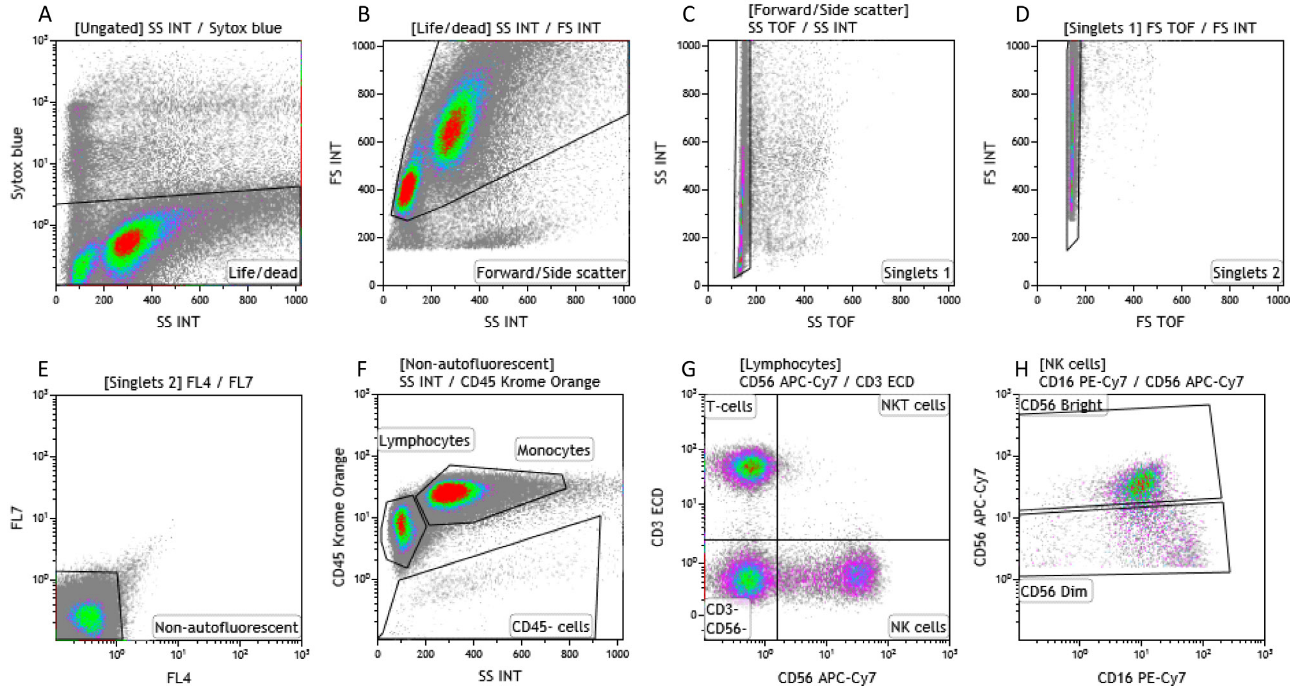

Supplementary Figure 1: Gating strategy for CD56+ NK cells and CD56 dim versus CD56 bright in ascites. In (**A**) and (**B**) life cells are gated, in (**C** and **D)** the single cells are selected and in (**E**) auto fluorescent cells are excluded from further analysis. In (**F**) the lymphocytes are gated. In (**G**) the CD56 positive cells are seen, with two populations. In (**H)** the NK CD56 bright and the NK CD56 dim are gated.
